# Synthesis of Short-Chain-Fatty-Acid Resveratrol Esters and Their Antioxidant Properties

**DOI:** 10.3390/antiox10030420

**Published:** 2021-03-10

**Authors:** You-Lin Tain, Sam K. C. Chang, Jin-Xian Liao, Yu-Wei Chen, Hung-Tse Huang, Yu-Lun Li, Chih-Yao Hou

**Affiliations:** 1 Department of Pediatrics, Kaohsiung Chang Gung Memorial Hospital and Chang Gung University College of Medicine, Kaohsiung 833, Taiwan; tainyl@hotmail.com; 2 Institute for Translational Research in Biomedicine, Kaohsiung Chang Gung Memorial Hospital and Chang Gung University College of Medicine, Kaohsiung 833, Taiwan; 3 Costal Research and Extension Center, Experimental Seafood Processing Laboratory, Mississippi State University, Pascagoula, MS 39567, USA; schang@fsnhp.msstate.edu; 4 Department of Food Science, Nutrition and Health Promotion, Mississippi State University, Mississippi State, MS 39762, USA; 5 Department of Seafood Science, National Kaohsiung University of Science and Technology, Kaohsiung 811, Taiwan; j0920181@gmail.com (J.-X.L.); yah880406@gmail.com (Y.-L.L.); 6 Department of Medicine, Chang Gung University, Linkow 333, Taiwan; naosa720928@gmail.com; 7 Department of Biochemical Science and Technology, National Taiwan University, Taipei 10617, Taiwan; kk49310953@nricm.edu.tw

**Keywords:** resveratrol esters, resveratrol, short-chain fatty acids, Steglich esterification, antioxidation

## Abstract

To expand the applications and enhance the stability and bioactivity of resveratrol (RE), and to simultaneously include the potential health benefits of short chain fatty acids (SCFA) esters of RE were prepared by Steglich reactions with acetic, propionic, and butyric acids, respectively. RE and the esterified RE-SCFA products (including RAE, RPE, and RBE) were analyzed using nuclear magnetic resonance (NMR), Fourier-transform infrared (FTIR) spectroscopy, thermogravimetric analysis (TGA), differential thermal analysis (DTA), and liquid chromatography–mass spectrometry (LC–MS). The FTIR and ^13^C NMR spectra of the esterified products included ester-characteristic peaks at 1751 cm^−1^ and 171 ppm, respectively. Moreover, the peaks in the range of 1700 to 1600 cm^−1^ in the FTIR spectra of the esterified products indicated that the esterification of RE-SCFA was successful. The TGA results revealed that the RE-SCFA esters decomposed at lower temperatures than RE. The peaks in the LC–MS profiles of the esterified products indicated the formation of mono- and diesters, and the calculated monoester synthesis rates ranged between 45.81 and 49.64%. The RE esters inhibited the Cu^2+^-induced low-density lipoprotein oxidation reaction, exhibited antioxidant activity in bulk oil, and effectively inhibited the hydroxyl radical-induced DNA scission. Moreover, the RE-SCFA esters had better hydrogen peroxide scavenging activity than RE. Our results are the first in the literature to successfully including short chain fatty acids in the esters of resveratrol, and the products could be used as a functional food ingredient in processed foods or can be used as dietary supplements to promote health.

## 1. Introduction

The synthesis of resveratrol derivatives and their novel functional properties have been a research hotspot recently. Resveratrol (RE; 3,4′,5 trihydroxystilbene) is a C6-C2-C6 stilbene natural phenolic compound with three hydroxyl groups [1] that is found in more than 70 plants and plays an important role in the defense against pathogens, infections, injury, and abiotic stress [1,2]. Resveratrol presents numerous therapeutic benefits, including anti-inflammatory, antioxidant, anti-platelet, anti-hyperlipidemia, immune-modulator, anti-carcinogenic, cardioprotective, vasorelaxant, and neuroprotective properties. In vitro, ex vivo, and animal studies have indicated that RE could present several health benefits, and cardiovascular protection [3,4,5]. However, many studies have demonstrated that resveratrol, similar to other polyphenols, presents very low bioavailability [6,7,8]. The low in vivo bioactivity of RE is one of its major drawbacks; however, it has been demonstrated that RE esterification could increase bioactivity [9]. RE as a natural phytoalexin with a stilbene moiety, which presents anti-inflammatory properties, is the most used template for drug design. Therefore, recently, technologies for the synthesis of RE derivatives have been developed; moreover, structure–activity relationship studies have revealed that the parent structure of RE is critical for its specific therapeutic effects [10]. The cytotoxicity of the RE derivative obtained by substituting the hydroxyl groups of RE with methoxy groups was higher than that of RE [11]; 4,4′-dihydroxy-trans-stilbene, an RE analog, with two hydroxyl groups in positions 4 and 4′ having higher anti-proliferative potential than RE [12]. Our research and others have shown that synthesized RE-butyric acid (RE-B) ester has a higher ability to decrease liver fat accumulation and antioxidant capacity than the resveratrol itself [9,13].

Recently, short-chain fatty acids (SCFA) have been proven to have benefits for human health. SCFAs are small organic monocarboxylic acids with two to six carbon chain lengths [14]. The most common SCFAs include acetic (C2), propionic (C3), and butyric (C4) acids. They are metabolites primarily produced by gastrointestinal bacteria and account for over 85% of the total SCFAs produced in the gut [15,16]. The SCFAs produced by gut microbes via fermentation of dietary polysaccharides, including fiber and resistant starch [14], can counteract systemic inflammatory and metabolic diseases, such as diabetic nephropathy. For example, mice deficient in the metabolite-sensing G protein-coupled receptor GPR43 or GPR109A were protected by SCFA (acetate, butyrate, or propionate) supplementation in the high-fiber diets afforded protection against development of kidney disease in diabetic mice [17]. Hu et al. (2020) indicated that SCFAs play a critical role in supporting β-cell metabolism and promoting survival under stressful conditions [18]. Therefore, supplementation with SCFAs, such as acetic, propionic, and butyric acids could benefit human health. In our previous study, a variety of RE derivatives were designed and synthesized using the Steglich esterification reaction, and we have observed the ability of resveratrol butyric ester (RBE) to prevent liver fat accumulation [9]. We have demonstrated that RBE outperformed RE in adjusting Acetyl-CoA carboxylase and SREBP-1 levels and reducing fat accumulation in HepG2 cells, when equal concentrations of RE and RBE were tested. These results indicated that the esterification of RE improved its biological activity. Research on simultaneous esterification of RE with the three common short chain fatty acids has not been reported.

Therefore, in this study, we aimed to synthesize RE-SCFA esters via the esterification of RE with acetic, propionic, and butyric acids, respectively, and to characterize the yields and physicochemical characteristics of the products by using nuclear magnetic resonance (NMR), Fourier-transform infrared (FTIR) spectroscopy, thermogravimetric analysis (TGA), differential thermal analysis (DTA), and liquid chromatography–mass spectrometry (LC–MS). Furthermore, a series of antioxidant and DNA mutation tests were performed to evaluate the bioactivity of the synthesized RE esters.

## 2. Materials and Methods

### 2.1. Materials

*Trans*-RE was purchased from TCI Development Co., Ltd. (Shanghai, China); acetic acid, propionic acid, and *n*-butyric acid were procured from ACROS (Madison, WI, USA); 1-ethyl-3-(3-dimethylaminopropyl) carbodiimide (EDC), and 4-dimethylaminopyridine (DMAP) were supplied by Sigma-Aldrich (St. Louis, MO, USA); tetrahydrofuran (THF) was supplied by Echo Chemical Co., Ltd. (Miaoli County, Taiwan); and Tris-acetate-EDTA (TAE) buffer was acquired from VWR International Inc. (West Chester, PA, USA).

### 2.2. Synthesis of RE-SCFA Esters

RBE was synthesized according to the modified method of Neises and Steglich [19], and Tain YL et al. [9]. A trans-RE (45.64 g, 0.2 mol) mixture with different individual SCFA including the acetic acid (19.38 g, 0.22 mol), propionic acid (19.38 g, 0.22 mol), and *n*-butyric acid (19.38 g, 0.22 mol), respectively, then was added to anhydrous THF (1000 mL) in a three-necked flask equipped with a magnetic stirrer. To perform the reaction in the dark, the flask was wrapped in aluminum foil. After the reactants were completely dissolved, EDC (34.16 g, 0.22 mol) and DMAP (13.44 g, 0.11 mol) were added to the solution. The esterification reaction was performed by stirring the solution at 28–30 °C under a nitrogen atmosphere for 48 h. Subsequently, the solution was poured into an excess amount of deionized water, and a viscous substance was precipitated. After the viscous product was re-dissolved in acetone and collected, the solvent was removed using a rotary vacuum evaporator. The resulting slurry of esters of RE-SCFA concentrate was frozen at −80 °C and freeze-dried, and the light yellowish dried product were placed in a brown vial to protect from potential light damage and stored in a refrigerator at 4 °C.

### 2.3. Physical Properties and Chemical Compositions of RE and RE-SCFA Esters

The methods used for physical characteristics of the RE-SCFA products, including that for FTIR, NMR (Both ^1^H NMR and ^13^C NMR analyses were performed using the Bruker AVANCE 600 MHz NMR spectrometer, Bruker (Billerica, MA, USA) with deuterated dimethyl sulfoxide (DMSO-*d*6) as the solvent at 30 °C), TGA analyses, and HPLC quantitation of the fatty acid esters of resveratrol were similar to that reported in our previous study [9].

### 2.4. Antioxidant Activity of RE-SCFA Esters in Bulk Oil

First, 1 g of antioxidant-free corn oil was added to a 10 mL flask followed by adding 100 μL of 75 μM RE or RE-SCFA ester solutions in ethanol to each sample. After the ethanol solution was blow-dried with liquid nitrogen, the flask was wrapped with aluminum foil to prevent light exposure. The flask was placed in an oven at 60 ± 0.5 °C and samples were collected after 0, 1, 3, and 6 days to analyze the conjugated diene content and determine the p-anisidine value according to the method of Faas et al. [20].

### 2.5. Antioxidant Activity of RE-SCFA Esters in Oil-in-Water Emulsion (β-Carotene Bleaching Assay)

The method of Zhong and Shahidi [21] was used to measure the antioxidant capacities of RE and the RE esters in oil-in-water emulsions utilizing the β-carotene bleaching assay. Each sample was dissolved in chloroform and was added to a 10 mL flask. Subsequently, 10 mg of β-carotene, 40 mg of linoleic acid, and 400 mg of Tween 40 were added to each sample. A control sample (without β-carotene) was prepared by mixing 40 mg of linoleic acid with 400 mg of Tween 40. After chloroform was purged with nitrogen gas, 100 mL of oxygenated distilled water was added to each sample and the mixtures were vigorously mixed. The absorbance of each sample was immediately measured at 470 nm. Subsequently, the mixtures were incubated in a thermostatic water bath at 50 °C for 105 min, and the absorbance at 470 nm was measured again. The antioxidant activities of RE and RE esters in the oil-in-water emulsions were calculated as follows:

Antioxidant activity (%) = [1 − (S_0_ − S_t_)/(C_0_ − C_t_)] × 100 (1)

where S_0_ and S_t_ are the absorbance of the test compound at 0 and 105 min, respectively, and C_0_ and C_t_ are the absorbance of the control at 0 and 105 min, respectively.

### 2.6. Ability of RE-SCFA to Inhibit Cu^2+^-Induced LDL Oxidation

Inhibition of Cu^2+^-induced low-density lipoprotein (LDL) oxidation was measured using the method described by Zhong and Shahidi [13]. All reagents were preheated at 37 °C for 15 min before adding 100 μL of 50 μM CuSO_4_ and 100 μL of 10 mM LDL to each sample. Immediately after the reagents were mixed, the initial absorbance was measured at 234 nm. After the samples were incubated at a constant temperature of 37 °C for 8 h, their absorbance at 234 nm was measured again to determine the formation of conjugated dienes. Background correction was subsequently performed by replacing LDL and CuSO_4_ with phosphate buffered saline (PBS) to calculate the inhibition rate.

### 2.7. Ability of RE-SCFA to Inhibit Hydroxyl Radical-Induced DNA Scission

The inhibitory activity of the RE-SCFA esters against hydroxyl radical-induced DNA scission was measured using the method of Oh and Shahidi [13] with the modifications described by Ambigaipalan and Shahidi [22]. pBR 322 DNA (50 μg/mL) was dissolved in 10 mM PBS (pH 7.4). Subsequently, RE and RE-SCFA esters dissolved in ethanol were diluted with PBS. For each test, the RE or RE-SCFA ester sample (12.5 μM, 2 μL), PBS (2 μL), DNA (50 μg/mL, 2 μL), FeSO_4_ (0.5 mM, 2 μL), and H_2_O_2_ (1 mM, 2 μL) were added to an Eppendorf tube. The control groups included a DNA + free radicals sample and a DNA only sample. After each tube was allowed to react at 37 °C for 1 h, 1 μL of dye (0.25% bromophenol blue, 0.25% xylene cyanol, and 50% glycerol) was added. The mixture was loaded onto 0.7% agarose gel (50 mL), which was prepared in Tris-acetate-EDTA (TAE) buffer (40 mM, pH 8.5) with SYBR dye (5 μL). Electrophoresis was performed at 80 V for 90 min in TAE buffer using an Electrophoresis System (Owl Separation Systems Inc., Portsmouth, NH, USA) horizontal micro-electrophoresis system with a 300 V power supply. DNA bands were observed under UV irradiation using an Alpha-Imager (Cell Biosciences, Santa Clara, CA, USA) gel recording system. The data were processed using the Chemi-Imager 4400 software.

### 2.8. Statistical Analyses

All analytical experiments were performed at least three times, and three samples were analyzed for each test. Data were collected and analyzed using one-way ANOVA and the Duncan’s test. Significant differences were set at *p* < 0.05. All statistical analyses were performed using the SPSS statistics software (version 12.0, St. Armonk, NY, USA) software.

## 3. Results

### 3.1. Synthesis of RE-SCFA Esters

**Figure 1:**
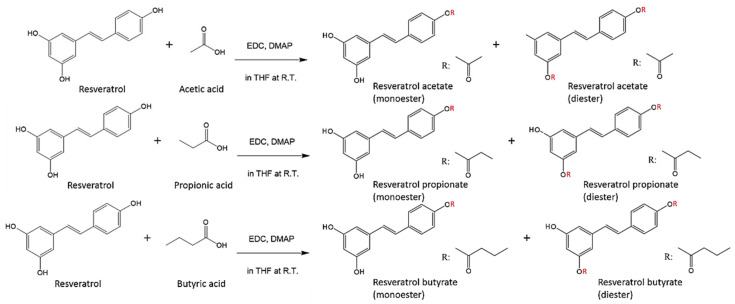
Reactions for the synthesis of resveratrol esters. EDC, 1-ethyl-3-(3-dimethylaminopropyl) carbodiimide; DMAP, 4-dimethylaminopyridine; THF, tetrahydrofuran; R.T., room temperature (28–30 °C).

Figure 1 shows the synthesis reactions of resveratrol with short chain fatty acids (Figure 1). Compared with traditional esterification, this ester coupling reaction, which was performed at near ambient temperature (28–30 °C) could prevent RE deactivation and degradation. In this study, we used water-soluble EDC instead of *N*,*N*′-dicyclohexylcarbodiimide (DCC), one of the most widely used activating and dehydrating agents for the Steglich esterification reaction, which was developed in 1978 by Neises and Steglich [19]. After 48 h, deionized water was added to stop the esterification reaction. EDC, DMAP, and the urea byproduct dissolved in water, the catalyst was disengaged during this step, and pale yellow RE-SCFA esters were obtained after freeze-drying.

### 3.2. Physical Properties and Chemical Composition of RE and RE-SCFA Esters

**Figure 2:**
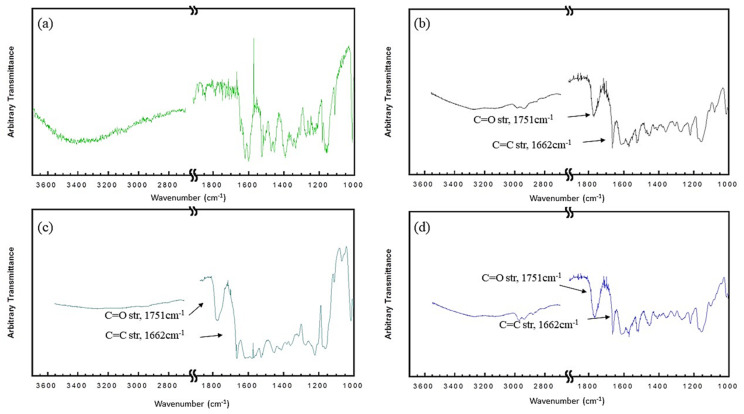
Fourier-transform infrared spectra of (**a**) resveratrol, (**b**) resveratrol acetate, (**c**) resveratrol propionate, and (**d**) resveratrol butyrate.

The FTIR spectra of RE, resveratrol acetic acid ester (RAE), resveratrol-propionic acid ester (RPE), and resveratrol butyric acid ester (RBE) are presented in Figure 2. The FTIR spectra of RE matched that reported by Porto et al. [23]. The broad band in the wavenumber range of 3400 to 3200 cm^−1^ in the spectrum of RE, which was assigned to the O–H stretching, was not present in the spectra of the esters. Conversely, a new absorption band at 1751 cm^−1^, which was ascribed to the ester C=O stretching, was observed in the spectra of RAE, RPE, and RBE. These results demonstrated that RE was successfully esterified via the coupling reaction with EDC. The assignments of the other characteristic bands of RE and its esters are illustrated in Figure 2. The bands at approximately 1600, 1510, and 1442 cm^−1^, which were attributed to the aromatic C=C stretching, were observed in all spectra. In addition, a small band at approximately 3020 cm^−1^, which was associated with the alkene and aromatic C-H vibrations, and a band at 1662 cm^−1^, which was ascribed to the alkene C=C stretching, were observed in all spectra. 

**Figure 3:**
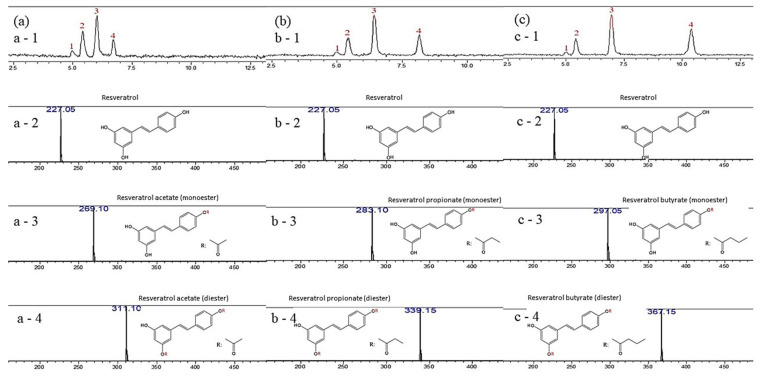
Liquid chromatography profiles of the products of the esterification of resveratrol with (**a**) acetic, (**b**) propionic, and (**c**) butyric acid. a-1, b-1, and b-3 represent the liquid chromatography analysis results of RAE, RPE, and RBE, respectively; a-2, a-3, and a-4 represent the MS assay result of liquid chromatography peaks 2, 3, and 4 of RAE. b-2, b-3, and b-4 represent the MS assay result of liquid chromatography peaks 2, 3, and 4 of RPE. c-2, c-3, and c-4 represent the MS assay result of liquid chromatography peaks 2, 3, and 4 of RBE.

The esterification of RE with acetic, propionic and butyric acids would likely yield three types of RE esters, namely mono-, di-, and triesters. In this study, LC–MS analysis was used to identify the contents of pristine RE, RAE, RPE, and RBE in the esterification products. The LC profiles in the negative ionization mode of RE and the esterification products showed four peaks (Figure 3). Peak 1 in the LC profiles could not be matched using the MS database. Peak 2 in the LC profiles of the esterification products, was observed at approximately 5.5 min, and matched the RE peak in the MS database. Peaks 3 and 4 in the LC profile of the product of the esterification of RE with acetic acid (Figure 3a) were detected at approximately 6.0 and 6.7 min, respectively, and were ascribed to the m/z values of RAE mono- (269.1) and diesters (311.1), respectively. Peaks 3 and 4 in the LC profile of the product of the esterification of RE with propionic acid (Figure 3b) were detected at approximately 7.0 and 8.2 min, respectively and were associated with the m/z values of RPE mono- (283.1) and diesters (339.15), respectively. Peaks 3 and 4 in the LC profile of the product of the esterification of RE with butyric acid (Figure 3c) were detected at approximately 7.0 and 10.4 min, respectively, and were attributed to the m/z values of RBE mono- (297.05) and diesters (367.15), respectively. In addition, the signals associated with the RE triesters were negligible (the value less than 1%). The LC–MS results revealed that the final products of the esterification of RE with acetic, propionic, and butyric acids were mixtures of RE and RAE, RPE, and RBE mono- and diesters, respectively. 

The products of the esterification with each short chain fatty acid of RE were shown in the Table 1. The esterification of RE with acetic acid consisted of RE (approximately 25.34%, underivatized), RAE monoester (49.64%), and RAE diester (23.40%). The products of the esterification of RE with propionic acid comprised RE (approximately 19.91%), RPE monoester (45.81%), and RPE diester (32.80%); and the products of the esterification of RE with butyric acid contained RE (approximately 17.11%), RBE monoester (47.12%), and RBE diester (35.00%) (Table 1). In our previous study, almost all RBE synthesized (reactions between RE and butyric acid) was in the monoester form, which suggested the synthesis duration may affect the composition of the final esterification product [9]. The quantity of RE-SCFAs obtained with Neises and Steglish esterification was 73.04–82.12% compared to the classical method of 37.7–74% [13] is more significantly improve. In addition, this method of esterification is more convenient in operation than the classical method, and it was easier to separate and obtain esterification products. Furthermore, Vitor Gilles et al. (2015) reported adding CeCl_3_ as catalysts in esterification could increase the yield pure monoesters to 50–56% [24], which suggest the process of esterification reaction still had the possibility of improving by modifying catalysis. In this study, the mono- and diesters appeared to be the main products of the esterification of RE with acetic, propionic, and butyric acids. Therefore, in future biological research the esterification products should be subjected to further chromatographic purification.

**Figure A1:**
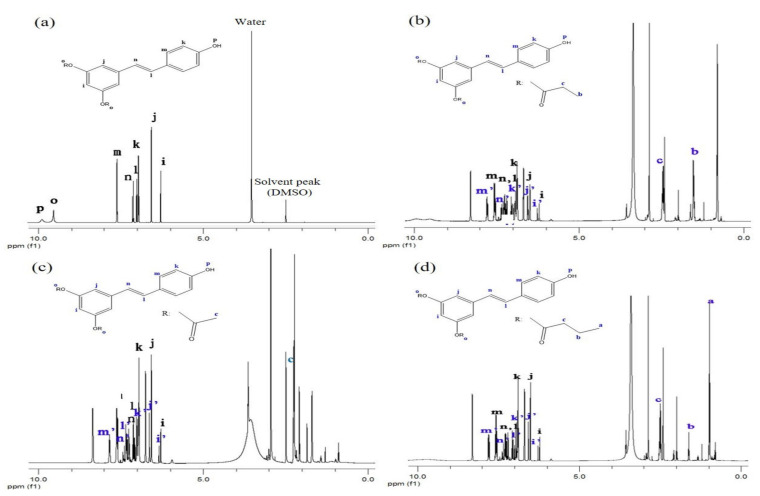
^1^H nuclear magnetic resonance spectra of (**a**) resveratrol, (**b**) resveratrol acetate, (**c**) resveratrol propionate, and (**d**) resveratrol butyrate. Here, DMSO Dimethyl sulfoxide.

**Figure A2:**
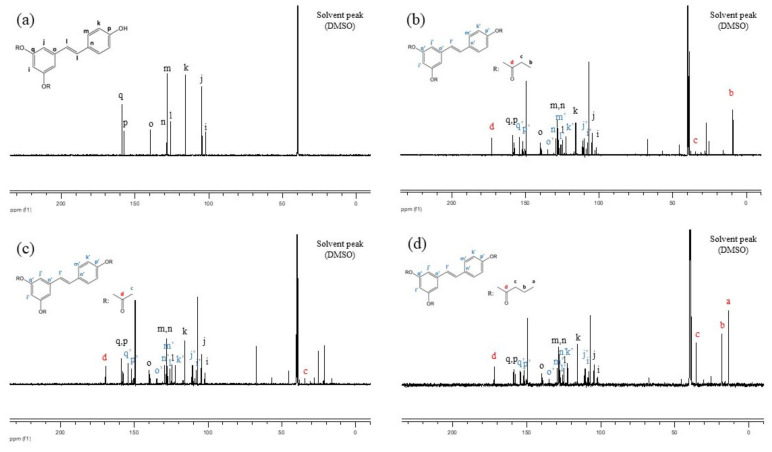
^13^C nuclear magnetic resonance spectra of (**a**) resveratrol, (**b**) resveratrol acetate, (**c**) resveratrol propionate, and (**d**) resveratrol butyrate. Here, DMSO Dimethyl sulfoxide.

The results of ^1^H NMR spectra from the NMR experiments confirming the chemical structures of the pristine RE, RAE, RPE, and RBE are presented in Figure A1. The results matched the spectral data reported by Lu et al. [25], which indicated that several signals appeared in the chemical shift range of 6–10 ppm after esterification. These signals were associated with RAE, RPE, and RBE (Figure A1b–d, respectively). However, not all the original signals of pristine RE shifted or disappeared from the spectra of the RE esters. These results were similar to our previous findings and suggested that the esterification products consisted of mixtures and esterification was not complete [9], which was consistent with the aforementioned LC–MS results. The ^13^C NMR spectra of pristine RE, RAE, RPE, and RBE are illustrated in Figure A2. The signal at 170 ppm corresponded to the C atoms of the ester carbonyl group, which indicated that esterification had occurred. Furthermore, additional peaks associated with pristine RE were detected in the ^13^C-NMR spectra of the reaction products. Our previous studies had indicated that the chemical structure of RE affected its biological activity [9]. In particular, the antioxidant properties of RE had been shown to correlate with the position of the hydroxyl groups [26]. In addition, the biological properties of RE had been expanded using several modifications, including hydroxylation, methylation, and isoprenylation, and also via the formation of di-, tri-, and oligomers [27]. Furthermore, the applications of RE and its derivatives had been expanded to the development of new anticancer agents. Oh and Shahidi [13] had synthesized 12 RE acyl chloride derivatives with different carbon chain lengths (C3:0-C22:6) and reported that the RC6:0, RC8:0, RC10:0, RC12:0, and RC16:0 derivatives presented better antioxidant activity than pristine RE in a bulk oil system. Moreover, the RC20:5n−3 and RC22:6n−3 esters presented the highest antioxidant activity in a meat model system (thiobarbituric acid reactive substance) when added to ground meat. Therefore, RE-SCFA showed higher antioxidant activity than the pristine RE in a bulk oil system and their activities depended on the esterification position, number of esterification substitutions, and polymer structure.

**Figure 4:**
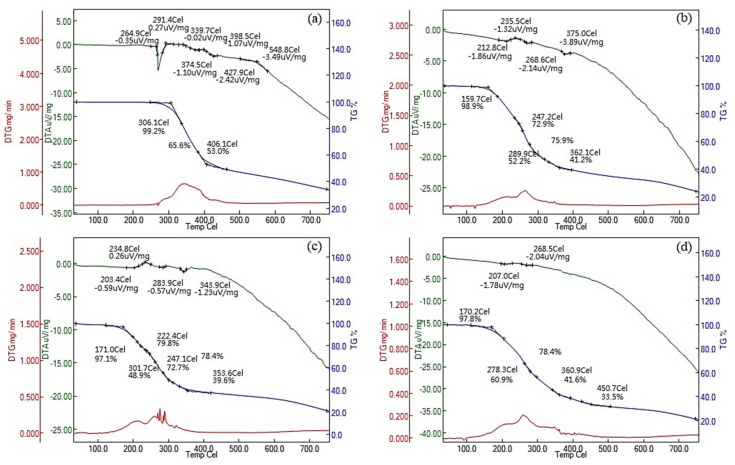
Thermograms of (**a**) pristine resveratrol (RE) and the products of the esterification of RE with (**b**) acetic, (**c**) propionic, and (**d**) butyric acids obtained under a nitrogen flow in the temperature range of 50–750 °C.

Thermogravimetric analysis/differential thermal analysis (TGA/DTA) experiments were performed to analyze the polymerization behavior of the RE-SCFA esters, and the results are presented in Figure 4. DTA is an analytical method that involves tracking mass changes in samples subjected to heating or cooling to induce physical or chemical changes. The most significant mass loss of RE was observed at 306.1 °C. The DTA curve of RE revealed a significant increase in mass loss rate starting at 260 °C, whereas the RE-SCFA esters lost mass in the temperature range of 159.7–171.0 °C. This was attributed to the decrease in thermal stability caused by structural changes after esterification. The main upward peak in the range of 170–200 °C in the DTA curves of the RE esters was attributed to the thermal decomposition of the esters. These results suggested that the RE-SCFA esters were thermally less stable than RE, a similar result also reported in the previous study which also indicated the RBE after esterification more thermo sensitivity [9].

### 3.3. Antioxidant Activity of RE Esters in Bulk Oil

Corn oil contains palmitic, linoleic, stearic, linolenic, and oleic acids [28], and can be easily oxidated to produce large peroxides and conjugated dienes. However, as the storage time of corn oil increases, it produces secondary oxidation products, such as aldehydes, furan derivatives, alcohols, ketones, and lactones. These oxidation products reduce the safety and nutritional value of oils [29]. Therefore, we used an antioxidant-free corn oil, which contained 34.1% oleic acid and 47.0% linoleic acid [30] to analyze the antioxidant capacity of RE and its derivatives. The conjugated diene content and p-anisidine values of corn oil samples with added RE, RAE, RPE, and RBE, and the results are summarized in Table 2. The conjugated diene content of all samples increased significantly from day 0 to day 1 and increases again on day 6. The p-anisidine values of the samples were not significantly changed on day 1 and day 3 but increased on day 6. These results trends were attributed to the absence of aldehydes, ketones, and quinones during the early oxidation stage. The p-anisidine value of the corn oil sample with added RPE increased significantly from day 3 to day 6, when it reached 27.89. On day 6, the p-anisidine value of the corn oil sample with added RAE was only 10.71, which indicated that RAE presented the best antioxidant capacity of all RE esters in this study. All RE esters inhibited the formation of conjugated dienes well, which was consistent with the results reported by Oh and Shahidi [13].

### 3.4. Antioxidant Activity of RE Esters in Oil-in-Water Emulsion (β-Carotene Bleaching Assay)

The β-carotene bleaching assay is one of the most popular methods for evaluating the antioxidant activity of compounds [31]. In this study, we used the method of Oh and Shahidi [13] to evaluate the antioxidant activity of RE and its esters in oil-in-water emulsions. Tween 40 was used to emulsify linoleic acid and form an oil-in-water emulsion. After β-carotene was added to the emulsion, linoleic acid oxidized and formed a peroxide upon heating the emulsion. Subsequently, β-carotene oxidation was induced, and the loss in the original orange-yellow color of β-carotene after oxidation was measured spectrophotometrically. The results indicated that the antioxidant capacity of RE was significantly higher than those of RAE, RPE, and RBE, which was ascribed to the substitution of the hydroxyl groups of RE with ester groups after esterification (Table 3). According to the polar paradox theory, non-polar antioxidants present better antioxidant effects than polar antioxidants in oil-in-water emulsions [32]. This could be attributed to oxidation mainly occurring at the emulsion surface. Polar antioxidants mainly reside in the aqueous phase of emulsions, whereas non-polar antioxidants are encapsulated in emulsions, which increases their antioxidant capacity. However, non-polar antioxidants should be surface-active to effectively remove metal ions and peroxides from the emulsion surface [33]. Although RAE, RPE, and RBE were less polar than RE, they lacked surface activity and, consequently they were unable to remove peroxides from the emulsion surface. Therefore, the antioxidant capacity of the RE-SCFA esters in oil-in-water emulsions was lower than that of RE.

### 3.5. Inhibition of Cu^2+^-Induced Low-Density Lipoprotein Oxidation

Low-density lipoprotein (LDL) oxidation has attracted the interest of researchers because oxidized LDL is a risk factor for atherosclerosis and coronary heart disease. In addition, low and high concentrations of oxidized LDL can induce inflammation and cell apoptosis, respectively [34]. Therefore, research on the inhibition of LDL oxidation has received considerable attention. In this study, LDL oxidation was induced using copper sulfate (CuSO_4_), and the conjugated diene content of the samples with added RE, RAE, RPE, and RBE was determined spectrophotometrically. RE presented pro-oxidant effects (Table 3). Dytrtová et al. [35] reported that the co-existence of RE and Cu^2+^ led to the formation of radicals with strong oxidative activity and a Cu^2+^-RE complex, which can intercalate DNA [36]. In this study, the substitution of hydroxyl groups of RE with ester groups led to changes in the sites that would react with Cu^2+^, and therefore, hindered oxidation. In addition, RAE, RPE, and RBE also inhibited Cu^2+^-induced LDL oxidation, and RPE showed the highest inhibition rate (79.0%) of all RE esters in this study.

### 3.6. Inhibition of Hydroxyl Radical-Induced DNA Scission

**Figure 5:**
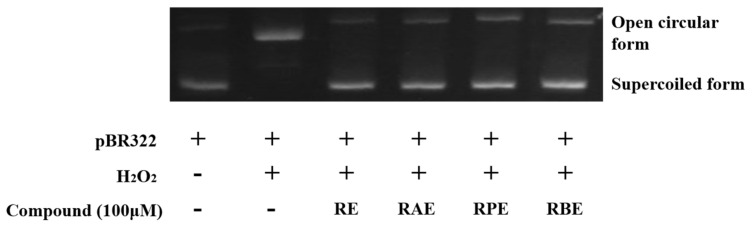
Inhibition of hydroxyl radical-induced DNA scission. Here, RE, RAE, RPE, and RBE denote resveratrol, resveratrol acetate, respectively.

DNA damage has always been considered a critical senescence driver, as the progressive loss of telomeric DNA over time causes different aging-related diseases. In particular, oxidative DNA damage has received significant attention from researchers. Cell metabolism and immune responses generate many reactive oxygen species, which cause oxidation of organic molecules (including DNA) in cells, and consequently, result in DNA damage-induced aging or mutations [37]. Therefore, numerous studies have investigated the use of plant-based antioxidants to prevent oxidative DNA damage [38,39,40]. In this study, we used hydrogen peroxide and Fe^2+^ ions to generate hydroxyl free radicals, which led to the conversion of linear DNA into circular DNA after the free radical attack. Almost all linear DNA molecules were converted to circular DNA after hydrogen peroxide was added (Figure 5). However, when RE and its esters were added to the pretreated DNA samples, DNA damage was significantly inhibited. Rossi et al. [41] investigated the protective effects of RE and its natural derivatives against oxidative DNA damage and indicated that RE presented protective effects via hydrogen atom donation, which scavenged free hydroxyl radicals. In addition, this study also indicated that, after esterification, the RE-SCFA esters also significantly inhibit DNA damage.

## 4. Conclusions

This study demonstrated the FTIR and NMR data were used for structure identification and to confirm the feasibility and stability of the synthesized products RE-SCFA esters (include RAE, RPE, and RBE). The TGA/DTA results revealed that the thermal resistance of the RE-SCFA esters was lower than that of RE, the monoester synthesis rates, which were calculated using the LC–MS peak area, ranged between 45.81% to 49.64%. Moreover, the RE-SCFA esters effectively inhibited β-carotene bleaching, Cu^2+^ induced LDL oxidation, and hydroxyl radical-induced DNA scission. These results indicated that RAE, RPE, and RBE, which were synthesized using the Steglich esterification method, can be used as functional food ingredients for processed foods or as health-promotion dietary supplements.

## Figures and Tables

**Table 1:** Resveratrol ester peak area ratios.

Sample	Peak 1 ^a^ (%)	Peak 2 ^b^ (%)	Peak 3 ^b^ (%)	Peak 4 ^b^ (%)
Resveratrol acetate	1.620	25.34	49.64	23.40
Resveratrol propionate	1.475	19.91	45.81	32.80
Resveratrol butyrate	0.768	17.11	47.12	35.00

^a^ peak 1 profile could not be matched using the MS database. ^b^ Peaks 2, 3, and 4 represent the contents of resveratrol, resveratrol monoester and resveratrol diester, respectively.

**Table 2:** Conjugated diene contents and p-anisidine values of stripped corn oil samples with added resveratrol and resveratrol esters over a 6-day period.

Sample	Conjugated Diene Content (%)				*p*-Anisidine Value			
	Storage Period				Storage Period			
	Day 0	Day 1	Day 3	Day 6	Day 0	Day 1	Day 3	Day 6
Resveratrol	0.09 ± 0.00 ^a^	0.16 ± 0.00 ^a^	0.16 ± 0.02 ^a^	0.28 ± 0.01 ^a^	8.71 ± 0.12 ^a^	4.29 ± 0.06 ^b^	4.69 ± 0.06 ^b^	16.40 ± 0.55 ^c^
Resveratrol acetate	0.07 ± 0.01 ^a^	0.14 ± 0.00 ^a,b^	0.14 ± 0.00 ^a,b^	0.27 ± 0.01 ^a,b^	3.93 ± 0.09 ^a^	4.79 ± 0.09 ^a^	4.41 ± 0.09 ^a^	10.71 ± 0.97 ^b^
Resveratrol propionate	0.05 ± 0.01 ^a^	0.16 ± 0.01 ^b^	0.17 ± 0.01 ^b^	0.29 ± 0.01 ^b^	0.27 ± 0.31 ^a^	4.13 ± 0.41 ^b^	9.19 ± 0.16 ^c^	27.89 ± 0.51 ^d^
Resveratrol butyrate	0.09 ± 0.06 ^a^	0.15 ± 0.01 ^b^	0.17 ± 0.00 ^b^	0.29 ± 0.00 ^b^	3.71 ± 0.03 ^a^	4.05 ± 0.10 ^a^	4.91 ± 0.07 ^a^	18.65 ± 1.53 ^b^

Data are presented as average ± standard deviation (*n* = 3). ^a–d^ Averages in the same row with different uppercase letters are significantly different (*p* < 0.05).

**Table 3:** Inhibitory effects of resveratrol and its esters against β-carotene bleaching and Cu^2+^-induced low-density lipoprotein (LDL) oxidation.

Sample	β-Carotene Bleaching (Inhibition %)	LDL (Inhibition %)
Resveratrol	60.9 ± 0.51 ^a^	−59.9 ± 4.62 ^b^
Resveratrol acetate	46.7 ± 1.01 ^b^	74.1 ± 33.2 ^a^
Resveratrol propionate	41.1 ± 0.54 ^d^	79.0 ± 11.3 ^a^
Resveratrol butyrate	44.9 ± 0.83 ^c^	64.8 ± 20.3 ^a^

Data are presented as average ± STD (*n* = 3). ^a–d^ Average in the same row with different uppercase letters are significantly different (*p* < 0.05).

## Data Availability

All data are constrained in the manuscript.
